# Rare Pseudosarcomatous Lesions Posing Diagnostic Challenges: Histopathologic Examination as a Dominant Tool Preventing Misdiagnosis of Proliferative Fasciitis

**DOI:** 10.7759/cureus.25770

**Published:** 2022-06-08

**Authors:** Andrej Ozaniak, Jiri Vachtenheim, Renata Chmelova, Robert Lischke, Zuzana Strizova

**Affiliations:** 1 Third Department of Surgery, First Faculty of Medicine, Charles University and Motol University Hospital, Prague, CZE; 2 Department of Pathology and Molecular Medicine, Second Faculty of Medicine, Charles University and Motol University Hospital, Prague, CZE; 3 Department of Immunology, Second Faculty of Medicine, Charles University, University Hospital Motol, Prague, CZE

**Keywords:** marginal excision, misdiagnosis, pseudosarcomatous lesion, soft tissue sarcoma, proliferative fasciitis

## Abstract

Proliferative fasciitis is an extremely rare benign myofibroblastic proliferation that typically presents as a rapidly growing subcutaneous mass. Precise histopathological interpretation is required to obtain a proper diagnosis. Due to a symptomatology overlap, discrimination from soft tissue sarcomas is crucial in the prevention of unnecessary excessive treatment that could be potentially harmful to the patients. Here, we present a rare case of atypical localization of proliferative fasciitis. The lesion was predominantly localized in the groin with the invasion of the scrotum and clinically mimicked soft tissue sarcoma. However, according to a proper histopathologic analysis, the diagnosis of proliferative fasciitis was concluded. With a large number of pseudosarcomatous lesions, there is a rising urge to introduce these rare but benign processes to physicians in order to prevent misdiagnosing patients.

## Introduction

Proliferative fasciitis and other pseudosarcomatous lesions are rare benign myofibroblastic proliferations that display similar clinical and histological features to soft tissue sarcomas [1-3]. Marginal excision is generally curative and the recurrence of the disease after proper surgical excision is rare [1,4]. Characteristic microscopic patterns, such as increased cellularity, nuclear pleomorphism, spindle-cell growth, and infiltrative growth represent a microscopic overlap with malignant soft tissue sarcomas. In addition, the pseudosarcomatous lesions contain polygonal cells that morphologically mimic ganglion cells. These ganglion-like elements may also be infrequently seen in rhabdomyosarcoma, ganglioneuroblastoma, and myxoinflammatory fibroblastic sarcoma [4-6]. The clinical manifestation of pseudosarcomatous lesions generally includes a rapidly growing and tender mass that arises in the upper or lower extremities. In a number of cases, pseaudosarcomatous lesions are of traumatic origin [4,7]. A recent study by Bala et al. demonstrated a rare case of pleomorphic lipoma which was initially presented as a cellular nerve sheath tumor [8]. In this pseudosarcomatous lesion, only a careful examination of characteristic histopathological features allowed reaching the correct diagnosis and prevented an overtreatment in the form of extensive surgery and additional therapies [8]. We present a case of an extremely rare localization of proliferative fasciitis. Because pseudosarcomatous lesions are prone to misdiagnoses, as seen in the study by Bala et al., we highlight that a standardized diagnostic approach, including proper histopathological examination, is crucial to avoid unnecessary and radical treatment for these benign lesions.

## Case presentation

**Figure 1:**
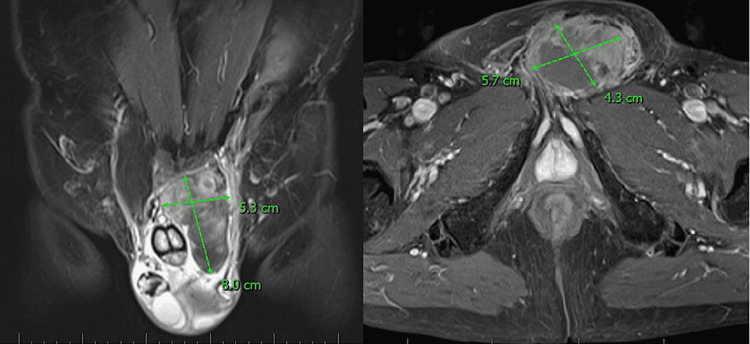
MRI finding of the initial lesion

We present a 70-year-old male patient who was originally reported as a suspected case of soft tissue sarcoma. The lesion presented as a palpable, fast-growing and mildly painful mass predominantly localized in the groin with the invasion of the scrotum. The skin above the lesion remained intact. To evaluate the extent of the lesion, an MRI scan with a contrast dye focused on the thigh, groin, and pelvis was performed (Figure 1).

**Figure 2:**
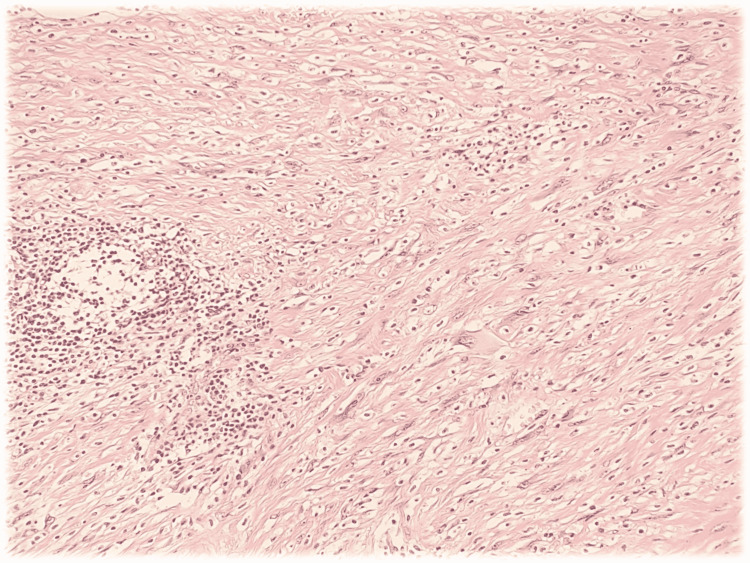
Histopathological image showing hematoxylin-eosin-stained tissue section: myofibroblastic cells with large nuclei and prominent nucleoli

As observed, the lobular tumorous expansion of 60x55x75mm in size was localized in the groin, contained both solid and cystoid components, and extended to the radix of the penis. The lesion was further ongoing to the upper end of the left part of the scrotum dislocating the spermatic cord laterally. However, signs of the infiltration of the spermatic cord or the surrounding tissues were not shown. Lymphadenopathy was ruled out. The next step included a 14G core needle biopsy (CNB). All samples displayed a proliferation of plump spindle fibroblastic/myofibroblastic cells with large nuclei and prominent nucleoli (Figure 2).

**Figure 3:**
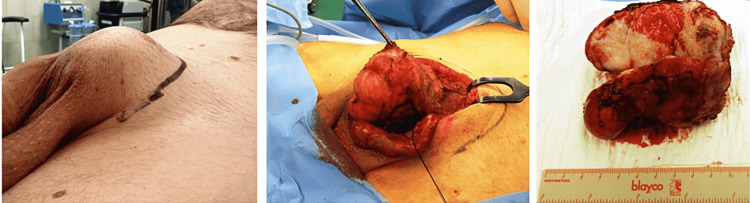
Marginal resection and macroscopic examination of the specimen

Among those cells, large cells with irregular shapes and abundant amphophilic to basophilic cytoplasm were patchily spread. These cells are known as ganglion-like elements and bear a singular nucleus or multiple large nuclei with only one prominent nucleolus [5]. Mitotic activity was not observed throughout the tissue samples. The stroma focally underwent myxoid and collagenous changes. Signs of focal necrosis with a mixed inflammatory infiltrate were seen and accented particularly in the perivascular areas of the lesion. Immunohistochemistry was performed on formalin-fixed and paraffin-embedded tissue samples. Spindle-cell proliferation showed positive staining for smooth muscle actin while the staining for desmin remained negative. The ganglion-like cells displayed only weak and focal positivity of smooth muscle actin and S100beta. The proliferation activity assessed by Ki-67 (MiB21) was minimal. According to the microscopical appearance and the immunohistochemical profile, the diagnosis of proliferative fasciitis was concluded. Since the diagnosis of soft tissue sarcoma was positively ruled out, a marginal local extirpation could be performed on the patient (Figure 3). During a 54-month postoperative follow-up, the patient has not shown any signs of local recurrence and remained disease- and pain-free.

## Discussion

Proliferative fasciitis is a benign pseudosarcomatous lesion that was shown to occasionally mimic soft tissue sarcoma [9]. Diagnostic errors occur frequently, and misdiagnosis for malignant neoplasm may further guide the inaccurate, and potentially harmful, treatment. On the other hand, owing to the rarity of soft tissue sarcomas, some of these lesions are often unknowingly resected without multi-disciplinary team involvement, which may increase the risk of residual tumor burden [10].

In our unusual case, a 70-year-old male patient with very atypical localization of proliferative fasciitis was presented. The palpable tumor mass appeared unusual throughout the clinical examination and the imaging techniques revealed a lobular tumorous expansion in the groin, resulting in an extensive debate on a possible malignant origin of such finding. The CNB was performed to avoid misinterpretation of the groin-localized process. The CNB, as well as incisional biopsy, is characterized by a high diagnostic accuracy and even though, discussions about the ideal biopsy techniques are still ongoing, CNB was shown to have lower risks of potential complications [11,12]. Therefore, in the deep-seated masses in the pelvic region, CNB should be performed preferentially [11,12]. In our case, the typical ganglion-like elements were microscopically present throughout all the bioptic samples. However, these patterns may also be infrequently seen in rhabdomyosarcoma, ganglioneuroblastoma, and myxoinflammatory fibroblastic sarcoma. According to the precise histopathologic evaluation, including assessment of both mitotic activity and proliferative index, as well as the smooth muscle actin and S100beta staining, the diagnosis of proliferative fasciitis was concluded and led to a marginal excision and a complete recovery of the patient.

## Conclusions

The diagnosis of pseudosarcomatous lesions can be challenging. Even though these lesions were previously demonstrated to hold characteristic microscopic features, and were associated with previous physical trauma, the rapid growth of pseudosarcomatous lesions may cause diagnostic confusion and subsequent treatment errors. It is of major importance to discriminate rare lesions, such as proliferative fasciitis, from soft tissue sarcomas to prevent unnecessary excessive treatment. Due to the histological overlap with other disease entities, proliferative fasciitis may be often regarded as malignant and accordingly overtreated. The knowledge of the specific pathologic features described is the best prevention of misdiagnoses. The estimation of the final diagnosis should, thus, depend on the evaluation of cytomorphological features and proper histopathological examination.
